# Probability Score to Predict Spontaneous Conversion to Sinus Rhythm in Patients with Symptomatic Atrial Fibrillation When Less Could Be More?

**DOI:** 10.3390/jcm13051470

**Published:** 2024-03-03

**Authors:** Marco Valerio Mariani, Nicola Pierucci, Sara Trivigno, Pietro Cipollone, Agostino Piro, Cristina Chimenti, Domenico Giovanni Della Rocca, Fabio Miraldi, Carmine Dario Vizza, Carlo Lavalle

**Affiliations:** 1 Department of Cardiovascular, Respiratory, Nephrological, Aenesthesiological and Geriatric Sciences, “Sapienza” University of Rome, 00161 Rome, Italy; nicola.pierucci@uniroma1.it (N.P.); sara.trivigno@uniroma1.it (S.T.); pietro.cipollone@uniroma1.it (P.C.); agostino.piro@uniroma1.it (A.P.); cristina.chimenti@uniroma1.it (C.C.); dario.vizza@uniroma1.it (C.D.V.); carlolavalle@yahoo.it (C.L.); 2 Texas Cardiac Arrhythmia Institute, St. David’s Medical Center, Austin, TX 78705, USA; domenicodellarocca@hotmail.it; 3 Cardio Thoracic-Vascular and Organ Transplantation Surgery Department, Policlinico Umberto I Hospital, 00161 Rome, Italy; fabio.miraldi@uniroma1.it

**Keywords:** atrial fibrillation, spontaneous conversion, emergency department

## Abstract

**Background:** The probability of spontaneous conversion (SCV) to sinus rhythm (SR) in patients presenting to the emergency department (ED) with hemodynamically stable, symptomatic atrial fibrillation (AF) is not well known. **Objective:** To develop and validate a score to determine the probability of SCV to SR in patients presenting to the ED with hemodynamically stable, symptomatic AF. **Methods:** This retrospective, observational study enrolled consecutive patients admitted with AF to the ED. Variables associated to SCV during a 6 h “wait-and-see” approach were used to develop and validate a score to determine the probability of SCV to SR in AF patients. The study was divided in two phases: (1) score development and (2) validation of the predictive score. **Results:** Out of 748 eligible patients, 446 patients were included in the derivation cohort, whereas 302 patients were included in the validation cohort. In the derivation cohort, based on multivariable logistic analysis, a probability score weight was developed including: previous SCV (3 points), AF-related symptom duration < 24 h (5 points), age ≥ 65 years (3 points) and female sex (2 points). The score allowed us to divide patients in three groups based on the probability of SCV to SR during the 6 h observation period. The probability prediction model showed an area under the curve (AUC) of 0.707 and 0.701 in the derivation and validation cohorts, respectively. **Conclusions:** The proposed score allowed us to predict SCV probability with good accuracy and may help physicians in tailoring AF management in an effective and timely manner.

## 1. Introduction

Atrial fibrillation (AF) is commonly encountered in daily clinical practice and is the most frequent arrhythmia leading to emergency department (ED) admission, accounting for up to 2% of all ED admissions [1]. Due to population-aging and the worldwide increase in prevalence of cardiovascular risk factors, AF prevalence is expected to grow in the foreseeable future, with a predicted number of patients suffering from AF of 17.9 million by 2060 [2]. In patients with hemodynamically stable symptomatic AF, current guidelines on AF management suggest a rhythm control strategy by pharmacological cardioversion and/or electrical cardioversion [3]. However, several studies have reported spontaneous conversion (SCV) rates of AF to sinus rhythm (SR) of up to 70% [4], leading physicians to consider a “wait-and-watch” strategy as a non-inferior alternative to early cardioversion in patients with recent-onset AF. The main challenge in the management of hemodynamically stable, symptomatic, recent-onset AF is moving from a “one-size-fits-all” approach to a “patient-centered” approach, tailoring the treatment strategy according to each patient’s probability of SCV to SR. On the one hand this new paradigm may reduce the overtreatment of patients who will likely convert spontaneously to SR, reducing cardioversion-related complications, and avoiding unnecessary hospitalizations and healthcare costs. On the other hand, the tailored approach may guarantee a timely cardioversion reducing the risk of negative outcomes and AF progression.

In this study we aimed to develop and validate a score to determine the probability of SCV in SR in patients presenting to the ED with hemodynamically stable, symptomatic AF.

## 2. Methods

### 2.1. Study Design and Population

This observational, retrospective study enrolled consecutive patients admitted with AF to the ED of Policlinico Umberto I Hospital of Rome from January 2020 to July 2023. Patients aged over 18 years, with hemodynamically stable, symptomatic AF were included. AF was electrocardiographically diagnosed either at ED admission or if the patient was admitted with an ECG showing AF obtained in an outpatient setting, even if the patient converted on his way to the hospital. Patients were treated following current European Society of Cardiology (ESC) guidelines on AF management [3], with either acute rate or rhythm control therapy applicable at the treating physician’s discretion. The following exclusion criteria were applied: hemodynamic instability, AF occurring in the context of critical illness (signs of heart failure [HF] or acute coronary syndrome), or persistent or permanent AF. Moreover, for the purpose of this study, we only included AF patients in whom a rhythm control strategy was not performed before presentation at the ED or within 6 h of ED admission, aiming to analyze variables associated with SCV. In order to develop and validate a score to determine the probability of SCV to SR in AF patients presenting to the ED, the study was divided in two phases: (1) score development in a derivation cohort of AF patients admitted to the ED from January 2020 to December 2021 and (2) validation of the predictive score in a set of consecutive AF patients admitted to the ED from January 2022 to July 2023. This study was approved by the Institutional Review Boards on Human Research.

### 2.2. Data Collection

The following baseline characteristics were collected: age, sex, presence of cardiovascular risk factors (such as systemic arterial hypertension, diabetes, dyslipidemia, smoking and familiar history of cardiovascular disease), history of coronary artery disease (CAD), chronic obstructive pulmonary disease (COPD), concomitant medications including antiarrhythmic and anticoagulant drugs, ECG features, laboratory investigation results and echocardiographic data. Additionally, we collected SCV occurrence, previous AF episodes and cardioversion attempts, previous SCV, the time of symptom/AF onset and treatments received as drugs for rate/rhythm control and cardioversion attempts. SCV was defined as conversion to SR without any active cardioversion attempt, either pharmacological cardioversion (PCV) or electrical cardioversion (ECV), before ED admission or during a 6 h observation in the ED. Rate-control strategy with beta blockers, verapamil and/or cardiac glycosides was attempted at the treating physician’s discretion and was not considered as active cardioversion attempt.

### 2.3. Statistical Methods

Categorical variables were expressed as numbers and percentages. Continuous variables were expressed as mean and standard deviation or median and interquartile range, as needed. The Kolmogorov–Smirnov test was used for the assessment of normal distribution of variables. Baseline demographic and clinical characteristics are presented in table format. Comparisons among variables were made using the Student’s *t*-test for normally distributed continuous variables, whereas categorical variables were compared using the χ2 test and the Fisher exact test. The Mann–Whitney U test was used to assess the differences between variables with a non-normal distribution. 

Univariate logistic regression analysis was performed to assess the association of the dependent variable SCV to SR with clinically plausible characteristics and all the variables with a significant association (*p*-value < 0.10) in the univariate analysis were included in a multivariable logistic model. The association between SCV to SR and the independent variables was expressed as odds ratio (OR) with a 95% confidence interval (95% CI). Model calibration was assessed by performing the Hosmer–Lemeshow goodness-of-fit, and variant inflation factors (VIFs) were used for multicollinearity diagnostics (a value of VIF < 3 for each variable was considered to exclude multicollinearity in the final model). Based on multivariable logistic analysis, a risk score weight was assigned to each significant predictor in the multivariable model by rounding the OR value to the next integer. The sum of the risk scores for each patient was calculated and the derivation cohort was divided into three groups based on the SCV probability. Risk score cut-offs were chosen with the aim of maximizing differences in SCV probability among the groups. Subsequently, the predictive score was tested in the validation cohort. The predictive performance of the score was assessed using the c-statistic in both the derivation and validation cohorts. For all tests, a *p*-value of less than 0.05 was considered statistically significant. Patients with missing data for any variable included in the predictive score were excluded from score development and validation analyses. The statistical analysis was performed using SPSS version 27.0 for Mac (IBM Software, Inc., Armonk, NY, USA). 

## 3. Results

Among 1057 consecutive patients admitted to the ED of Policlinico Umberto I Hospital of Rome between January 2020 to July 2023, 748 patients with hemodynamically stable and symptomatic AF were considered eligible for analysis. Out of 308 not-included patients, 35 were excluded due to hemodynamic instability and/or concomitant critical illness, 1 patient was under 18 years of age, 61 patients suffered from persistent or permanent AF, and in the last 212 patients, active cardioversion strategy was attempted before a 6 h “wait-and-see” approach due to treating physician’s preference (excluded patients‘ characteristics are described in Table S1). Out of 748 eligible patients, 446 patients (46.6% female; mean age 69 ± 13.8 years) admitted to the ED between January 2020 and December 2021 were included in the derivation cohort, whereas 302 patients (35.1% female; mean age 65.9 ± 15.1 years) admitted from January 2022 to July 2023 were included in the validation cohort (Table S2). An overview of the characteristics of study population is presented in Table 1. Overall, 300 (40.1%) patients spontaneously converted to SR during the 6 h observation period, 176 patients (39.5%) in the development cohort and 124 patients (41.1%) in the validation cohort, respectively. Among 270 patients in the development cohort who did not spontaneously convert to SR, PCV was attempted in 197 patients (73%), ECV was performed in 40 patients (14.8%), whereas in the last 33 patients (12.2%), a rate-control strategy was adopted. The overall success rate of cardioversion was 87% (206 out 237 patients). In the validation cohort, 28 out 178 patients (15.7%) who did not spontaneously convert to SR after a 6 h observation period were treated with a rate-control strategy, 123 patients (69.1%) underwent PCV, and the last 27 patients (15.2%) underwent ECV, with an overall success rate of cardioversion of 86% (129 out 150 patients). No thromboembolic events were observed.

### 3.1. Derivation of the Predictive Score

As shown in Table 1, in the derivation cohort, patients who converted spontaneously to SR were more likely female and older (*p*-value 0.012 and *p*-value 0.014, respectively), to have experienced a previous SCV (*p*-value < 0.001) and to report a shorter duration of AF-related symptoms (*p*-value < 0.001) than patients who did not convert. Moreover, the prevalence of chronic oral anticoagulant therapy (OAT) was larger among patients who did not convert spontaneously (*p*-value 0.009). As shown in Table S3, chronic OAT was associated with history of active cardioversion (*p*-value < 0.001) and with chronic antiarrhythmic therapy (*p*-value < 0.001).

**Figure 1:**
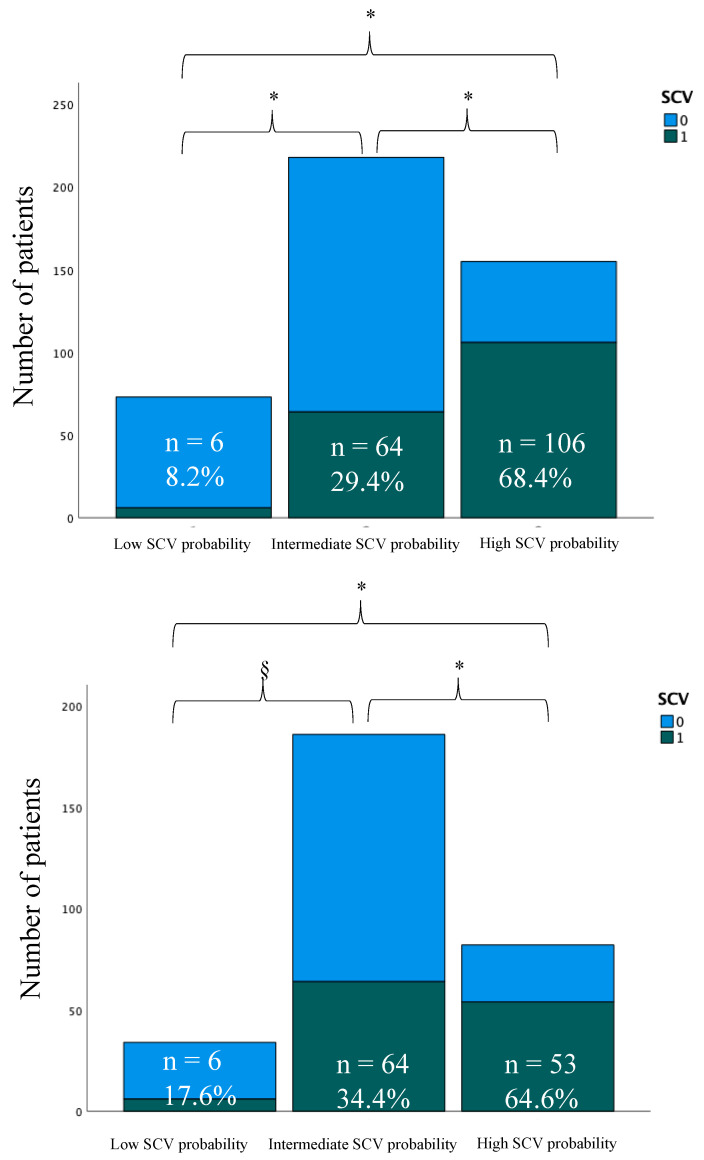
Observed incidence of spontaneous conversion across low (0 to 5), intermediate (6 to 9) and high (10 to 13) probability categories for spontaneous conversion (SCV) in the derivation cohort (superior panel) and the validation cohort (inferior panel). Numbers and percentages of patients experiencing SCV are shown, with *p*-values for comparisons of SCV rates among different groups. The symbol * indicates a *p*-value < 0.05, whereas the symbol § indicates a *p*-value ≥ 0.05.

At univariate analysis, four independent clinical variables were significantly associated with SCV and were included in the multivariable model (Table 2). None of the variables included in the final model showed multicollinearity (VIF < 3). Based on multivariable logistic analysis, a risk score weight was assigned to each significant predictor in the multivariable model by rounding the OR value to the next integer. In particular, the weighted score included: previous SCV (3 points), AF-related symptom duration < 24 h (6 points), age ≥ 65 years (2 points) and female sex (2 points). The weighted score allowed us to divide patients in three groups on the basis of probability of SVC to SR during the 6 h observation period (Figure 1): SCV rate in patients with score ranging from 0 to 5 was 11.3% (6/73 patients, low SCV probability group), whereas patients with score between 6 and 9 had a SCV probability of 29.4% (64/218 patients, intermediate SCV probability group) and a score from 10 to 13 was associated with an SCV probability of 68.4% (106/155 patients, high SCV probability group). A significant difference in SCV rates among groups was found, with an overall *p*-value < 0.001. The weighted score showed a significant association with the variable SCV, with an odds ratio associated with every increase of one score point of 1.373 (95% CI 1.231–1.532, *p*-value < 0.001).

**Figure 2:**
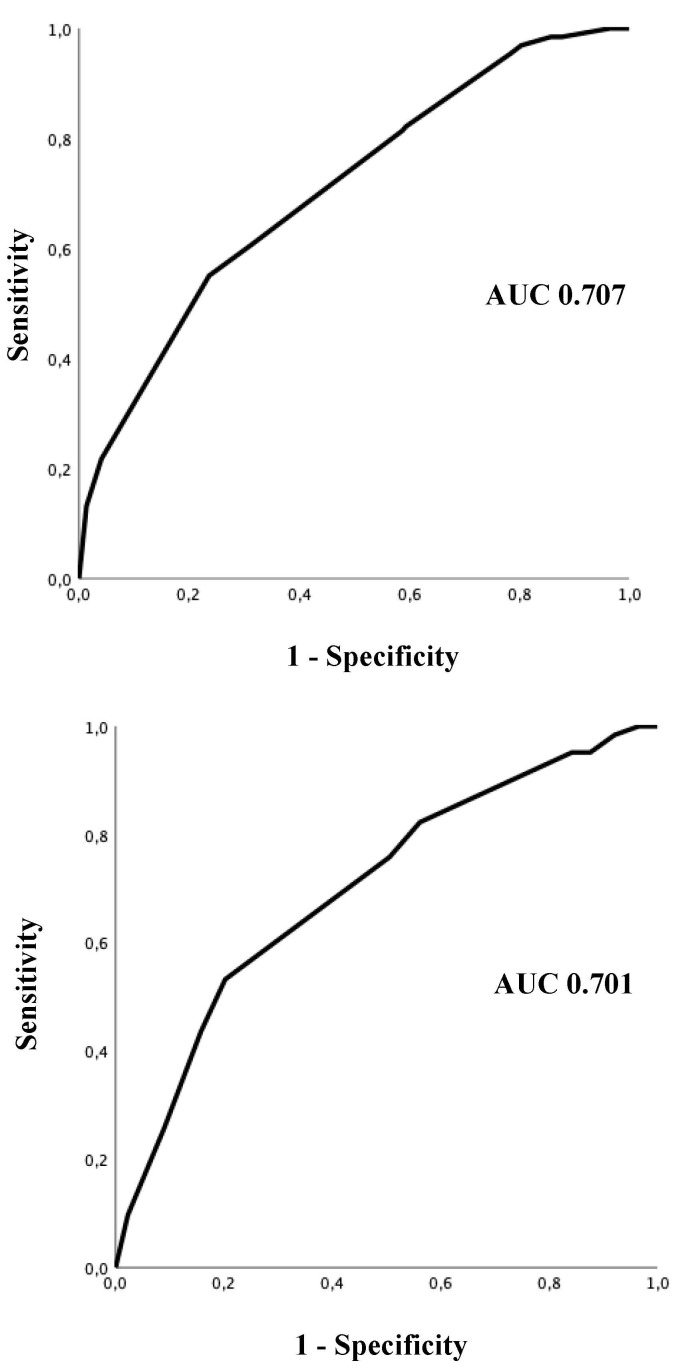
Receiver-operating characteristic (ROC) curve of probability score as a diagnostic test for the prediction of spontaneous conversion in the derivation cohort (superior panel) and the validation cohort (inferior panel). AUC: area under the curve.

The probability prediction model showed an area under the curve (AUC) of 0.707 (95% CI 0.646–0.767), and the *p*-value of the Hosmer–Lemeshow goodness-of-fit test was 0.443 (Figure 2).

### 3.2. Validation of the Predictive Score

In the validation cohort, 124 patients spontaneously converted to SR during the 6 h observation period. Among the patients in the low SCV probability group (score ≤ 5), 17.6% spontaneously cardioverted within 6 h of observation (6/34 patients), whereas 65.9% of patients classified in the high SCV probability group (score ≥ 10) experienced SCV to SR (54/82 patients). The rate of SCV in the intermediate SCV probability group (score 6–9) was 34.4% (64/186 patients), in line with the findings in the development cohort (Figure 1). A significant difference in SCV rates among groups was found, with an overall *p*-value < 0.001. As shown in Figure 2, the score showed good discrimination power with an AUC of 0.701 (95% CI 0.641–0.761).

## 4. Discussion

Herein we report a new probability score to predict SCV to SR in a real-world cohort of patients presenting with hemodynamically stable, symptomatic AF in the ED. The main findings of our analysis were as follows:

- SCV to SR of symptomatic AF during a 6 h observational period in the ED was relatively high (40.1%, 300 patients), suggesting that an active cardioversion strategy may not have been required in almost a half of patients;
- Independent predictors of SCV to SR included previous SCV, AF-related symptom duration < 24 h, age ≥ 65 years and female sex;
- The weighted probability score predicted SCV to SR during a 6 h observation in ED with reasonable accuracy, both in the derivation cohort (AUC 0.707) and in the validation cohort (AUC 0.701);
- The score divided patients in three groups based on the SCV probability: low probability group (score 0 to 5) of patients who are unlikely convert to SR (SCV rate ≅ 10%), intermediate probability group (score 6 to 9) with 1/3 of probability of SCV during a 6 h observation period and a high probability group (score 10 to 13), with a SCV rate as high as 68%.

Overall, 300 (40.1%) patients spontaneously converted to SR during the 6 h observation period, 176 patients (39.5%) in the development cohort and 124 patients (41.1%) in the validation cohort, respectively. Our results are in line with previous findings and with the concept that longer observation times are associated with higher SCV rate. Pluymaekers et al. [5] reported a rate of SCV to SR of 16.8% during an observation period of 3 h. In a randomized, controlled trial testing the effectiveness and safety of propafenone compared to a placebo in patients with recent-onset AF, Boriani et al. [6] reported a SCV rate of 37% in the placebo group during an 8 h observation period. Interestingly, SCV rate increased to 56% in the subgroup analysis, including patients without heart disease [6]. More recently, the randomized controlled RACE 7 ACWAS trial compared a “wait-and-see” approach with immediate cardioversion in patients presenting with recent-onset AF, and did not show a difference in terms of SR rate after 4 weeks of follow-up (91% vs. 94%, respectively). Of note, SCV occurred in 69% of patients during the 48 h observation period [7]. 

### 4.1. Predictors of SCV Included in the Probability Score

Independent predictors of SCV to SR during the 6 h observation period were previous SCV, AF-related symptom duration < 24 h, age ≥ 65 years and female sex. We described, for the first time, the history of previous SCV as an independent predictor of SCV to SR in the ED. This finding seems in line with the concept that fibrotic atrial cardiomyopathy (FACM) is the essential substrate for the onset and maintenance of AF, with progressive electroanatomic remodeling that perpetuates the arrhythmia, reducing the possibility of SCV [8]. Hence, patients who experiment with SCV probably have a less diseased and remodeled atrial substrate, likely resulting in higher probability of SCV. Conversely, as shown by Shaji et al. [9], history of previous ECV strongly reduces SCV probability, suggesting an underlying more advanced FACM. In line with previous results, AF-related symptoms with a duration < 24 h resulted the strongest SCV predictor in our weighted probability score. In 356 patients with AF of <72 h duration, Danias et al. [10] identified presentation < 24 h from symptoms onset as the only predictor of SCV, with significant association among SCV rate and total symptom duration (66% for duration < 24 h vs. 17% for duration > 48 h). Similarly, Niederdockl et al. [11] found AF-related symptom duration < 24 h was the strongest predictor of SCV in the ReSinus score. This result is probably related to the correlation between atrial effective refractory period (AERP) and AF duration, with median fibrillation interval and AERP shortening during the first 24 h of AF, promoting the electrophysiologic conditions for AF maintenance in long-lasting episodes, as shown by Wijffels et al. in a goat model of atrial fibrillation [8]. Interestingly, age ≥ 65 years was associated with a three-fold increase in SCV probability. In contradiction to the conventional wisdom about the association between age and atrial fibrosis, in autoptic investigations, Platonov et al. [12] did not find any correlation between patient age and fibrosis extent but found a correlation between fibrosis extent and AF presence, duration and severity. Furthermore, Mahnkopf et al. [13] found that the degree of left atrial structural remodeling was completely independent of co-existing cardiovascular co-morbidities, with the majority of patients with the so-called “lone AF” displaying chronic fibrotic bi-atrial substrate, despite the absence of apparent structural heart disease. Conversely, genetics and additional pathophysiological factors, as such inflammatory processes, seem to play a key role in FACM, that is considered a progressive disease where AF is a manifestation of the structural atrial cardiomyopathy [14]. Although the clinical manifestation of FACM with AF usually occurs in advanced ages, it might be speculated that when AF occurs in younger patients it may be associated with a more advanced fibrotic cardiomyopathy, resulting in a lower probability of SCV. In our study, female gender was associated with a two-fold increase in SCV probability compared to male sex. Similarly, Choudhary et al. [15] reported a higher SCV rate among female patients and related this result to a lower atrial fibrillatory rate (AFR) found in females compared to males. Despite the fact that sex difference in AF epidemiology is well-established, little is known about sex differences in mechanisms leading to AF maintenance and SCV probability. Previous studies have reported a shorter AERP and an attenuation of the arrhythmia-induced shortening of AERP in pre-menopausal women, suggesting an estrogen-mediated role for the lower AF incidence found among young women [16]. However, more recent studies did not find any sex-based difference in AERP, and hormone replacement therapy, compared to a placebo, did not reduce AF incidence in post-menopausal women [17]. Recently, Thibault et al. [18] compared atrial electrical and structural properties in male and female mice without AF, reporting no sex differences in action potential configuration, ionic currents or atrial collagen content. Moreover, they found higher atrial mass in males than females, due to larger size of male myocytes, and a more pronounced lateralization of the cellular distribution of the connexins Cx40 and Cx43, promoting non-linear conduction that may predispose to AF onset and maintenance. Noteworthy, they demonstrated that orchiectomy-related reduction in atrial cell size and connexin lateralization was associated with reduced AF susceptibility, whereas ovariectomy did not affect AF susceptibility. These results showed that a putative-favoring role of androgens in AF pathogenesis is hypothesis-generating and may explain our finding of higher SCV probability associated with female sex. 

### 4.2. Clinical Perspectives 

AF is a worrisome global health problem due to its impact on patients’ morbidity and mortality, with a prevalence estimated to double in the next decades [1]. Currently, AF accounts for about 2% of total admittance to the ED [1]. Current guidelines on AF management recommend a rhythm control strategy by PCV and/or ECV in patients presenting with hemodynamically stable, symptomatic AF [3]. However, active cardioversion attempts by means of PCV and/or ECV may be time- and resource-consuming, requiring long observation times and the involvement of different specialists, such as cardiologists, anesthesiologist and a dedicated echocardiographist performing a transesophageal echocardiogram (TOE). As a result, AF management in ED often leads to ward admission, with a rate of hospitalization as high as 38.3%, even though more than 1/3 of these patients are asymptomatic for AF [19]. Since the largest part of AF-related financial burden is attributable to inpatient stays and drug usage, a cost-effective AF management in the ED seems outstandingly important. A “wait-and-see” approach has been proposed as an alternative strategy to active cardioversion in patients with recent-onset AF presenting at the ED, with the aim of reducing hospital accesses and financial burden on health system. However, the “wait-and-see” approach with delayed conversion, as proposed by the RACE 7 ACWAS trial [7], may not be as cost-effective as previously thought. Indeed, patients who had undergone delayed cardioversion had more outpatient visits and often a second ED admission if not spontaneously converted, leading to a reduced efficiency, increased costs and more complicated treatment pathways [7]. Therefore, an appropriate identification of factors associated with SCV of AF to SR may help clinicians in selecting patients suitable for a “wait-and-see” approach and early ED discharge rather than PCV and/or ECV and hospitalizations, increasing system efficiency. 

Our weighted probability score predicted SCV to SR during a 6 h observation in ED with reasonable accuracy and may allow tailoring of AF management in each patient based on the probability of SCV, answering the question “When less could be more?”. Indeed, the score allows the identification of the subgroup of patients with higher SCV probability who may really benefit from a “wait-and-see” approach, compared to the low SCV probability group where a passive approach may be detrimental, thus paving the background for AF management optimization. This personalized approach may reduce PCV/ECV, and its possible complications, in patients with high SCV probability, and may identify patients with a low probability of SCV for whom a timely active conversion attempt should be pursued to reduce the risk of AF progression and related complications. Of note, the score is based on clinical predictors, thus it does not require laboratory exams or an echocardiogram, and may be calculated not only in the ED, but also by the cardiologist or general practitioner during an outpatient visit, possibly reducing ED admissions in patients with a high probability of SCV to SR. However, before our score is implemented in clinical practice, our results need to be confirmed in larger, external, validation cohorts. In recent years, artificial intelligence (AI) and machine learning (ML) systems have been extensively used to advance our understanding of AF, broadly in relation to AF detection, risk prediction and management [20]. In some cases, ML models were used for predicting factors related to AF management. For instance, Vinter et al. [21] developed a sex-specific prediction model for identifying patients who would benefit from ECV without AF recurrences. However, to date, no studies have evaluated the use of AI/ML systems to predict AF SCV in the ED setting. In this regard, the use of AI/ML-based algorithms are welcomed as they may improve our SCV probability prediction.

## 5. Limitations

Our study had several limitations. To begin with, the single-center design limited the generalizability of our results, and these need to be confirmed and validated in larger, external cohorts. Furthermore, given the retrospective nature of the study, AF management was not standardized and was left to the treating physician’s discretion. Eventually, it is conceivable that patients in the non-SCV cohort would have converted spontaneously with longer observation times. Hence, some bias cannot be excluded, and our results need to be interpreted with caution. 

## 6. Conclusions

Our weighted probability score predicted SCV to SR of hemodynamically stable, symptomatic AF during a 6 h observation period in ED with reasonable accuracy and may allow for tailoring of AF management in each patient based on the probability of SCV. This personalized approach may reduce PCV/ECV and hospitalizations in patients with high SCV probability, and may allow a timely cardioversion in patients with low SCV probability. Larger prospective studies are required to further validate this score as a stratification tool for AF SCV probability.

## Figures and Tables

**Table 1:** Demographics and baseline characteristics of the derivation and the validation cohort.

	Development Cohort (*n* = 446)			Validation Cohort (*n* = 302)			
	SCV (*n* = 176)	Non-SCV (*n* = 270)	*p*-Value	SCV (*n* = 124)	Non-SCV (*n* = 178)	*p*-Value	*p*-Value among Cohorts
**Clinical, ECG and Echo Characteristics**							
**Age, years (IQR)**	72.5 (15.8)	70 (20)	0.014	70 (24)	64 (19.8)	<0.001	0.002
**Female, *n* (%)**	95 (54%)	113 (41.9%)	0.012	56 (45.2%)	50 (28.1%)	0.002	0.002
**Heart rate, bpm (IQR)**	115 (37)	115 (35.3)	0.721	120 (50)	120 (40)	0.424	0.347
**LVEF, % (IQR)**	55 (5)	55 (5)	0.058	55 (4)	55 (5)	0.003	0.993
**TAPSE, mm (IQR)**	18 (4)	19 (5)	0.582	21 (3)	21 (4)	0.119	<0.001
**IVC, mm (IQR)**	14.5 (3)	15 (3)	0.112	15 (2)	15 (2)	0.546	<0.001
**Comorbidities**							
**HTN, *n* (%)**	116 (65.9%)	173 (64.1%)	0.692	90 (72.6%)	104 (58.4%)	0.012	0.875
**DM, *n* (%)**	24 (13.6%)	33 (12.2%)	0.662	14 (11.3%)	14 (7.9%)	0.313	0.138
**Dyslipidemia, *n* (%)**	56 (31.8%)	85 (31.5%)	0.940	52 (41.9%)	54 (30.3%)	0.038	0.320
**Current smoker, *n* (%)**	20 (11.4%)	19 (7%)	0.114	12 (9.7%)	26 (14.6%)	0.204	0.090
**Family history of CVD, *n* (%)**	24 (13.6%)	31 (11.5%)	0.499	18 (14.5%)	24 (13.5%)	0.828	0.529
**HF, *n* (%)**	15 (8.5%)	35 (13%)	0.146	12 (9.7%)	10 (5.6%)	0.182	0.074
**IHD, *n* (%)**	24 (13.6%)	46 (17%)	0.334	18 (14.5%)	27 (15%)	0.876	0.767
**Previous TIA/stroke, *n* (%)**	1 (0.6%)	2 (0.7%)	0.827	1 (0.8%)	1 (0.6%)	0.796	0.986
**COPD, *n* (%)**	8 (4.5%)	18 (6.7%)	0.350	4 (3.2%)	6 (3.4%)	1	0.114
**VHD, *n* (%)**	15 (8.5%)	19 (7%)	0.563	12 (9.7%)	18 (10.1%)	0.901	0.268
**AF history**							
**First AF episode, *n* (%)**	92 (52.3%)	128 (47.4%)	0.315	56 (45.2%)	72 (40.4%)	0.415	0.062
**Previous SCV, *n* (%)**	57 (32.4%)	37 (13.7%)	<0.001	56 (45.2%)	42 (23.6%)	<0.001	<0.001
**Previous ECV/PCV, *n* (%)**	0 (0%)	179 (66.3%)	<0.001	0 (0%)	74 (41.6%)	<0.001	<0.001
**Previous AF ablation, *n* (%)**	2 (1.1%)	5 (1.9%)	0.552	1 (0.8%)	3 (1.7%)	0.511	0.784
**AF symptoms < 24 h, *n* (%)**	122 (94.6%)	115 (77.7%)	<0.001	110 (88.7%)	140 (78.7%)	0.023	0.361
**AF symptoms duration, h (IQR)**	4 (5)	9.5 (20)	<0.01	8.5 (10)	8 (15)	0.853	<0.001
**CHA2DS2VASc score (IQR)**	3 (2)	2 (2)	0.031	3 (3)	2 (3)	0.001	0.022
**HAS-BLED (IQR)**	2 (1)	2 (1)	0.027	2 (2)	1 (2)	0.002	0.657
**Medication**							
**Oral anticoagulant therapy, *n* (%)**	48 (27.3%)	106 (39.3%)	0.009	33 (26.6%)	58 (32.6%)	0.266	0.209
**VKA, *n* (%)**	5 (2.8%)	7 (2.6%)	1	2 (1.6%)	4 (2.2%)	0.489	0.538
**LMWH, *n* (%)**	7 (4%)	13 (4.8%)	0.676	2 (1.6%)	0 (0%)	0.089	0.002
**NOAC, *n* (%)**	43 (24.2%)	99 (36.7%)	0.007	31 (25%)	54 (30.3%)	0.310	0.281
**Beta blockers, *n* (%)**	72 (40.9%)	123 (45.6%)	0.334	34 (27.4%)	38 (21.3%)	0.223	<0.001
**ACE blocker/AT-2 blocker, *n* (%)**	70 (39.8%)	90 (33.3%)	0.166	51 (41.1%)	65 (36.5%)	0.417	0.480
**MRA, *n* (%)**	6 (3.4%)	18 (6.7%)	0.136	6 (4.8%)	8 (4.5%)	0.889	0.649
**SGLT2i, *n* (%)**	14 (7.9%)	21 (7.8%)	0.946	12 (9.7%)	18 (10.1%)	0.901	0.320
**Statin, *n* (%)**	56 (31.8%)	70 (25.9%)	0.177	48 (38.7%)	50 (28.1%)	0.052	0.218
**Amiodarone, *n* (%)**	6 (3.4%)	10 (3.7%)	0.870	0 (0%)	6 (3.4%)	0.085	0.204
**Flecainide, *n* (%)**	18 (10.2%)	42 (15.6%)	0.107	10 (8.1%)	20 (11.2%)	0.365	0.147
**Propafenone, *n* (%)**	4 (2.3%)	10 (3.7%)	0.397	2 (1.6%)	2 (1.1%)	1	0.112
**Laboratory**							
**Hemoglobin, g/dL (IQR)**	13.8 (2.2)	14 (2.3)	0.113	12.6 (2.2)	12.6 (2)	0.737	<0.001
**Creatinine, mg/dL (IQR)**	0.9 (0.3)	1 (0.3)	0.017	0.8 (0.2)	0.8 (0.1)	0.175	<0.001
**Potassium, mmol/L (IQR)**	4 (0.6)	3.9 (0.9)	0.656	4 (0.2)	4 (0.3)	0.769	0.395
**Sodium, mmol/L (IQR)**	140 (4)	140 (4)	0.565	140 (3)	140 (4)	0.657	0.155
**hs-Troponin T, mcg/L (IQR)**	0.013 (0.014)	0.015 (0.022)	0.126	0.015 (0.022)	0.001 (0.019)	0.085	<0.001
**D Dimer, mg/dL (IQR)**	413.5 (455.3)	380 (460)	0.741	321 (182)	321 (46.8)	0.868	0.043
**LDH, U/L (IQR)**	197 (49)	197 (59.8)	0.836	222 (34)	224 (32)	0.079	<0.001
**CRP, mg/dL (IQR)**	0.24 (0.55)	0.21 (0.68)	0.785	0.1 (0.46)	0.1 (0.05)	0.168	<0.001

ACE: angiotensin converting enzyme; AF: atrial fibrillation; AT: angiotensin; COPD: chronic obstructive pulmonary disease; CRP: C-reactive protein; CVD: cardiovascular disease; DM: diabetes mellitus; ECV: electrical cardioversion; HF: heart failure; hs: high-sensitivity; HTN: hypertension; IHD: ischemic heart disease; IQR: interquartile range; IVC: inferior vena cava; LDH: lactate dehydrogenase; LMWH: low-molecular-weight heparin; LVEF: left ventricular ejection fraction; MRA: mineral corticoid receptor antagonist; NOAC: novel oral anticoagulants; PCV: pharmacological cardioversion; SCV: spontaneous conversion; SGLT2i: sodium–glucose cotransporter-2 inhibitors; TAPSE: tricuspid annular plan excursion; TIA: transient ischemic attack; VKA: vitamin K antagonist.

**Table 2:** Univariable and multivariable regression analyses for predictors of SCV to sinus rhythm.

Variables	Univariable			Multivariable		
	OR	95% CI	*p*-Value	OR	95% CI	*p*-Value
**Previous SCV**	3.016	1.887–4.821	<0.001	2.802	1.479–5.307	0.002
**Symptom duration < 24 h**	5.001	2.128–11.753	<0.001	5.772	2.377–14.019	<0.001
**Age > 65 years**	1.786	1.159–2.754	0.009	2.145	1.201–3.831	0.010
**Female sex**	1.630	1.112–2.389	0.012	1.819	1.063–3.113	0.029

CI: confidence interval; OR: odds ratio; SCV: spontaneous conversion.

## Data Availability

The data presented in this study are available on request from the corresponding author. The data are not publicly available due to privacy restrictions.

## Supplementary Materials

The following supporting information can be downloaded at: https://www.mdpi.com/article/10.3390/jcm13051470/s1. Table S1: Demographics and baseline characteristics of derivation cohort in according to age, sex and baseline oral anticoagulation therapy (OAT); Table S2: Flow Chart of included and excluded patients in the study; Table S3. Demographics and baseline characteristics of derivation cohort in according to age, sex and baseline oral anticoagulation therapy (OAT).
