# Supplementary material for: Probability Score to Predict Spontaneous Conversion to Sinus Rhythm in Patients with Symptomatic Atrial Fibrillation When Less Could Be More?
Source: J Clin Med. 2024 Mar 3;13(5):1470. doi: 10.3390/jcm13051470 (PMC10934271; doi:10.3390/jcm13051470)
Supplement: Supplementary file 1 [file jcm-13-01470-s001.zip › jcm-2870174-supplementary.pdf]

SUPPLEMENTARY MATERIAL

	Included Population (n=748)	Excluded population (n=221)	p-value
**Clinical, ECG and Echo Characteristics**			
Age, years (IQR)	69 (20)	69 (20)	0.701
Female, n (%)	314 (42%)	84 (38%)	0.291
Heart rate, bpm (IQR)	120 (40)	140 (20)	0.159
LVEF, % (IQR)	55 (5)	55 (6)	0.176
TAPSE, mm (IQR)	20 (7)	20 (6)	0.077
IVC, mm (IQR)	15 (2)	15 (2)	0.921
**Comorbidities**			
HTN, n (%)	483 (64.6%)	152 (69%)	0.247
DM, n (%)	85 (11.4%)	18 (8%)	0.172
Dyslipidemia, n (%)	247 (33%)	66 (30%)	0.377
Current smoker, n (%)	77 (10.3%)	29 (13%)	0.158
Family history of CVD, n (%)	97 (13%)	33 (15%)	0.316
HF, n (%)	72 (9.6%)	15 (7%)	0.194
IHD, n (%)	115 (15.4%)	32 (14.7%)	0.744
Previous TIA/stroke, n (%)	5 (0.7%)	3 (1.5%)	0.319
COPD, n (%)	36 (4.8%)	15 (7%)	0.248
VHD, n (%)	64 (8.6%)	18 (8%)	0.846
**AF history**			
First AF episode, n (%)	348 (46.5%)	91 (41%)	0.160
Previous SCV, n (%)	192 (25.7%)	60 (27.5%)	0.659
Previous ECV/PCV, n (%)	253 (33.8%)	88 (40%)	0.101
Previous FA ablation, n (%)	11 (1.5%)	2 (1.1%)	0.520
AF symptoms < 24 h, n (%)	487 (65.1%)	128 (58%)	0.051
AF symptoms duration, h (IQR)	7 (12)	7 (11)	0.114
CHA2DS2VASc score (IQR)	2 (3)	2 (3)	0.098
HAS-BLED (IQR)	2 (1)	2 (1)	0.979
**Medication**			

Oral anticoagulant therapy, n (%)	245 (32.8%)	84 (38%)	0.147
VKA, n (%)	18 (2.4%)	7 (3%)	0.530
LMWH, n (%)	22 (2.9%)	7 (3%)	0.862
NOAC, n (%)	227 (30.4%)	80 (36%)	0.100
Beta blockers, n (%)	267 (35.7%)	62 (28%)	**0.035**
ACE blocker/AT-2 blocker, n (%)	276 (36.9%)	86 (39%)	0.586
MRA, n (%)	38 (5.1%)	18 (8%)	0.086
SGLT2i, n (%)	65 (8.7%)	12 (5.3%)	0.115
Statin, n (%)	224 (29.9%)	77 (34.7%)	0.167
Amiodarone, n (%)	22 (2.9%)	10 (4.6%)	0.247
Flecainide, n (%)	90 (12%)	33 (15%)	0.255
Propafenone, n (%)	18 (2.4%)	7 (3%)	0.530
**Laboratory**			
Hemoglobin, g/dl (IQR)	13.3 (2.4)	13.3 (2.6)	0.166
Creatinine, mg/dl (IQR)	0.9 (0.2)	0.9 (0.2)	0.252
Potassium, mmol/l (IQR)	4 (0.35)	4 (0.33)	0.719
Sodium, mmol/l (IQR)	140 (2)	140 (2)	0.600
hs-Troponin T, mcg/l (IQR)	0.014 (0.019)	0.014 (0.018)	0.838
D Dimer, mg/dl (IQR)	350 (233)	343 (235)	0.703
LDH, U/l (IQR)	207 (52)	205 (53)	0.755
CRP, mg/dl (IQR)	0.13 (0.44)	0.13 (0.42)	0.074

Table S1. Demographics and baseline characteristics of derivation cohort in according to age, sex and baseline oral anticoagulation therapy (OAT)

ACE:angiotensin converting enzyme; AF:atrial fibrillation; AT:angiotensin; COPD:chronic obstructive pulmonary disease; CRP:C-reactive protein; CV:cardioversion; CVD: cardiovascular disease; DM:diabetes mellitus; ECV:electrical cardioversion; HF: heart failure; hs:high-sensitive; HTN: hypertension; IHD:ischemic heart disease; IQR: interquartile range; IVC: inferior vena cava; LDH:lactate dehydrogenase; LMWH: low-molecular weight heparin; LVEF: left ventricular ejection fraction; MRA: mineralcorticoid receptor antagonist; NOAC:novel oral anticoagulants; PCV: pharmacological cardioversion; SCV:spontaneous conversion; SGLT2i: sodium-glucose cotransporter-2 inhibitors; TAPSE: tricuspid annular plan excursion;TIA:transient ischemic attack; VKA: vitamin K antagonist.

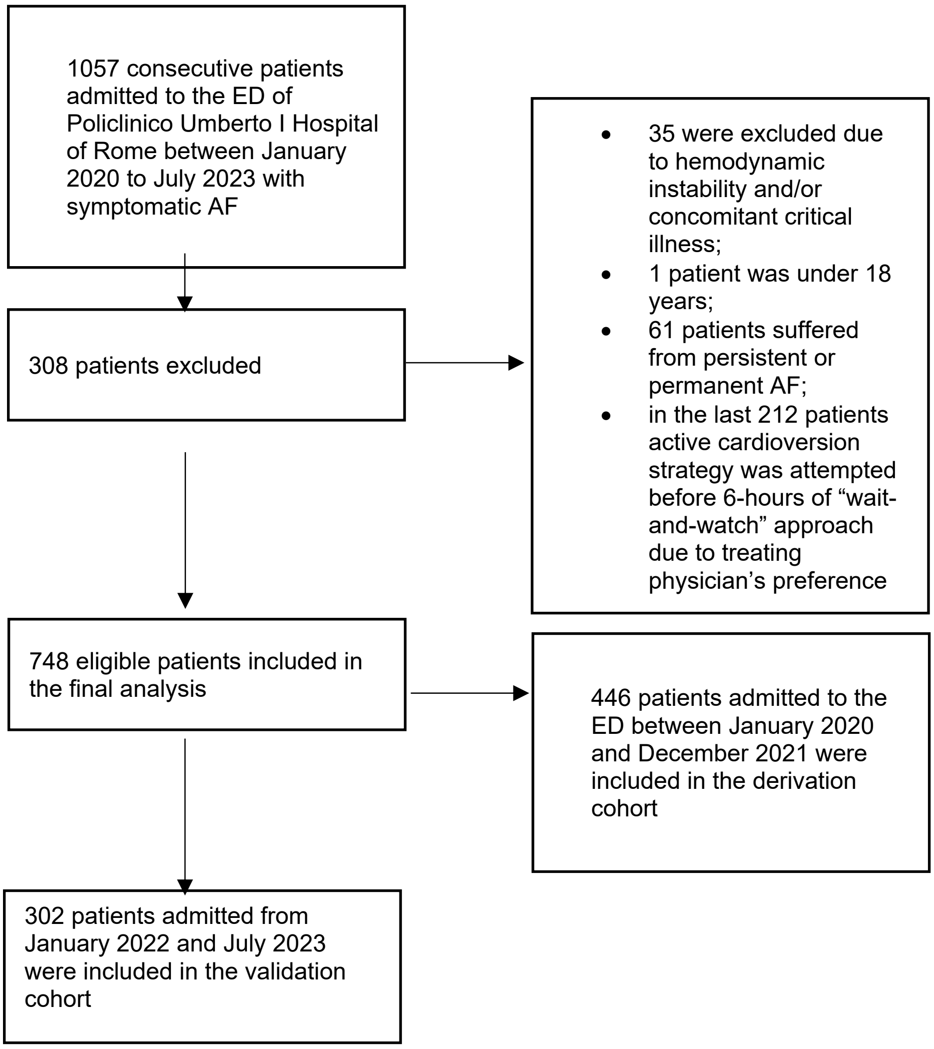

Table S2. Flow Chart of included and excluded patients in the study.

AF: atrial fibrillation; ED: emergency department

	Age (n=446)			Anticoagulation Status (n=446)			Sex (n=446)		
	< 65 years (n=133)	≥65 years (n=313)	p-value	Off chronic OAT (n=273)	On chronic OAT (n=173)	p-value	Male (n=238)	Female (n=208)	p-value
**Clinical, ECG and Echo Characteristics**									
Age ≥65 years, n (%)				164 (60.1%)	149 (86.1%)	**<0.001**	139 (58.4%)	174 (83.7%)	**<0.001**
Female, n (%)	34 (25.6%)	174 (55.6%)	**<0.001**	116 (42.5%)	92 (53.2%)	**0.027**			
Heart rate, bpm (IQR)	120 (40)	115 (35)	0.200	120 (30)	110 (30)	0.093	110 (32.5)	120 (40)	**0.040**
LVEF, % (IQR)	55 (5)	55 (5)	**<0.001**	55 (5)	55 (5)	**0.002**	55 (1)	55 (5)	0.296
TAPSE, mm (IQR)	19 (3)	18 (5)	0.068	19 (6)	18 (5)	0.111	19 (5)	18 (5)	0.732
IVC, mm (IQR)	15 (4)	15 (4)	0.471	14 (4)	15 (4)	0.766	15 (4)	15 (4)	0.349
**Comorbidities**									
HTN, n (%)	50 (37.6%)	239 (76.4%)	**<0.001**	150 (54.9%)	139 (80.3%)	**<0.001**	140 (58.8%)	149 (71.6%)	**0.005**
DM, n (%)	11 (8.3%)	46 (14.7%)	0.063	31 (11.4%)	26 (15%)	0.258	30 (12.6%)	27 (13%)	0.906
Dyslipidemia, n (%)	20 (15%)	121 (38.7%)	**<0.001**	75 (24%)	66 (38.2%)	**0.018**	65 (27.3%)	76 (36.5%)	**0.037**
Current smoker, n (%)	13 (9.8%)	26 (8.3%)	0.616	26 (8.3%)	13 (7.5%)	0.464	22 (9.2%)	17 (8.2%)	0.690

Family history of CVD, n (%)	13 (9.8%)	42 (13.4%)	0.284	37 (13.5%)	18 (10.4%)	0.324	28 (11.8%)	27 (13%)	0.696
HF, n (%)	8 (6%)	42 (13.4%)	**0.023**	20 (7.3%)	30 (17.3%)	**0.001**	35 (14.7%)	15 (7.2%)	**0.012**
IHD, n (%)	11 (8.3%)	59 (17.9%)	**0.005**	18 (6.6%)	52 (30.1%)	**<0.001**	49 (20.6%)	21 (10.1%)	**0.002**
Previous TIA/stroke, n (%)	1 (0.8%)	2 (0.6%)	0.893	2 (0.7%)	1 (0.6%)	0.845	2 (0.8%)	1 (0.5%)	0.643
COPD, n (%)	3 (2.3%)	23 (7.3%)	**0.036**	14 (5.1%)	12 (6.9%)	0.427	11 (4.6%)	15 (7.2%)	0.244
VHD, n (%)	14 (10.5%)	20 (6.4%)	0.132	18 (6.6%)	16 (9.2%)	0.303	19 (7.9%)	15 (7.2%)	0.759
**AF history**									
SCV, n (%)	40 (30.1%)	136 (43.5%)	**0.008**	121 (44.3%)	55 (31.8%)	**0.008**	81 (34%)	95 (45.7%)	**0.012**
First AF episode, n (%)	67 (50.4%)	153 (48.9%)	0.773	181 (66.3%)	39 (22.5%)	**<0.001**	121 (50.8%)	99 (47.6%)	0.494
Previous SCV, n (%)	31 (23.3%)	63 (20.1%)	0.451	46 (14.7%)	48 (27.7%)	**0.006**	51 (21.4%)	43 (20.7%)	0.845
Previous ECV/PCV, n (%)	77 (57.9%)	102 (32.6%)	**<0.001**	96 (30.7%)	83 (48%)	**0.007**	112 (47.1%)	67 (32.2%)	**0.001**
Previous FA ablation, n (%)	3 (2.3%)	4 (1.2%)	0.989	1 (0.4%)	6 (3.5%)	**0.010**	5 (2.1%)	2 (1%)	0.334
AF symptoms < 24 h, n (%)	79 (59.4%)	158 (50.5%)	0.918	150 (54.9%)	87 (79.1%)	**0.013**	130 (85%)	107 (86.3%)	0.755

AF symptoms duration, h (IQR)	5 (12.7)	6 (16.5)	0.797	5 (9)	8 (21)	**0.002**	5 (21)	6.5 (9)	0.508
CHA2DS2VASc score (IQR)	1 (2)	3 (2)	**0.001**	2 (2)	3 (2)	**<0.001**	2 (2)	3 (1)	**0.001**
HAS-BLED (IQR)	0 (1)	2 (1)	**0.001**	1 (2)	2 (1)	**<0.001**	1 (2)	2 (1)	**<0.001**
**Medication**									
Oral anticoagulant therapy, n (%)	24 (18%)	149 (47.6%)	**<0.001**				81 (34%)	92 (44.2%)	**0.027**
VKA, n (%)	1 (0.8%)	11 (3.5%)	0.119	0 (0%)	12 (6.9%)	**<0.001**	6 (2.5%)	6 (2.9%)	0.813
LMWH, n (%)	5 (3.8%)	15 (4.8%)	0.630	1 (0.4%)	19 (11%)	**<0.001**	10 (4.2%)	10 (4.8%)	0.758
NOAC, n (%)	19 (14.3%)	123 (39.3%)	**<0.001**	0 (0%)	142 (82.1%)	**<0.001**	66 (27.7%)	76 (36.5%)	**0.046**
Beta blockers, n (%)	38 (28.6%)	157 (59.2%)	**<0.001**	88 (32.2%)	107 (61.8%)	**<0.001**	95 (39.9%)	100 (48.1%)	0.083
ACE blocker/AT-2 blocker, n (%)	42 (31.6%)	118 (37.7%)	0.217	89 (32.6%)	71 (41%)	0.070	80 (33.6%)	80 (38.5%)	0.518
MRA, n (%)	4 (3%)	20 (6.4%)	0.148	11 (4%)	13 (7.5%)	0.112	10 (4.2%)	14 (6.7%)	0.238
SGLT2i, n (%)	10 (7.5%)	25 (8%)	0.866	23 (8.4%)	12 (6.9%)	0.568	19 (7.9%)	16 (7.7%)	0.909
Statin, n (%)	36 (27.1%)	90 (28.8%)	0.717	65 (23.8%)	61 (35.3%)	**0.009**	64 (26.9%)	62 (29,8%)	0.495
Amiodarone, n (%)	1 (0.8%)	15 (4.8%)	**0.036**	4 (1.5%)	12 (6.9%)	**0.002**	8 (3.4%)	8 (3.8%)	0.784
Flecainide, n (%)	14 (10.5%)	46 (14.7%)	0.238	17 (6.2%)	43 (24.9%)	**<0.001**	32 (13.4%)	28 (13.5%)	0.996

Propafenone, n (%)	3 (2.3%)	11 (3.5%)	0.486	10 (3.7%)	4 (2.3%)	0.425	7 (2.9%)	7 (3.4%)	0.798
**Laboratory**									
Hemoglobin, g/dl (IQR)	15 (2)	13.4 (2.1)	**<0.001**	14.2 (2.3)	13.5 (2.3)	**<0.001**	14.8 (2.1)	13.2 (1.8)	**<0.001**
Creatinine, mg/dl (IQR)	0.9 (0.2)	0.9 (0.3)	0.475	0.9 (0.2)	0.9 (0.3)	**0.019**	1 (0.2)	0.8 (0.3)	**<0.001**
Potassium, mmol/l (IQR)	140 (3)	140 (4)	0.367	140 (4)	140 (4)	**0.027**	140 (3)	140 (4)	0.243
Sodium, mmol/l (IQR)	3 (0.64)	4 (0.66)	**0.017**	3.9 (0.7)	4 (0.57)	0.720	3.99 (0.69)	3.91 (0.65)	0.211
hs-Troponin T, mcg/l (IQR)	0.011 (0.011)	0.016 (0.021)	**<0.001**	0.013 (0.016)	0.019 (0.021)	**0.002**	0.013 (0.014)	0.015 (0.020)	0.071
D Dimer, mg/dl (IQR)	319 (260)	446 (551)	**0.004**	441 (478)	373 (304)	0.323	313 (353)	480 (497)	**<0.001**
LDH, U/l (IQR)	191 (66)	198 (53)	0.485	194 (52)	200 (59)	0.484	192 (50)	201 (65)	**0.003**
CRP, mg/dl (IQR)	0.15 (0.42)	0.25 (0.73)	**0.014**	0.21 (0.56)	0.23 (0.6)	0.508	0.17 (0.59)	0.24 (0.55)	0.167

Table S3. Demographics and baseline characteristics of derivation cohort in according to age, sex and baseline oral anticoagulation therapy (OAT)

ACE:angiotensin converting enzyme; AF:atrial fibrillation; AT:angiotensin; COPD:chronic obstructive pulmonary disease; CRP:C-reactive protein; CV:cardioversion; CVD: cardiovascular disease; DM:diabetes mellitus; ECV:electrical cardioversion; HF: heart failure; hs:high-sensitive; HTN: hypertension; IHD:ischemic heart disease; IQR: interquartile range; IVC: inferior vena cava; LDH:lactate dehydrogenase; LMWH: low-molecular weight heparin; LVEF: left ventricular ejection fraction; MRA: mineralcorticoid receptor antagonist; NOAC:novel oral anticoagulants; PCV: pharmacological cardioversion; SCV:spontaneous conversion; SGLT2i: sodium-glucose cotransporter-2 inhibitors; TAPSE: tricuspid annular plan excursion;TIA:transient ischemic attack; VKA: vitamin K antagonist.
